# The Impact of Enforcement Capabilities on the Effectiveness of Public Assessment on Occupational Safety

**DOI:** 10.3390/ijerph17176426

**Published:** 2020-09-03

**Authors:** Manuel Soriano-Serrano, Jesús Antonio Carrillo-Castrillo, Juan Carlos Rubio-Romero, Manuel García-Jiménez

**Affiliations:** 1 Occupational Risk Prevention, Avenida Antonio Pascual Acosta, n° 1, 23009 Jaén, Spain; manuel.soriano.serrano@juntadeandalucia.es; 2 School of Industrial Engineering, Camino de los Descubrimientos sn, Universidad de Sevilla, 41092 Sevilla, Spain; 3 School of Industrial Engineering, Calle Doctor Ortiz Ramos (Teatinos) s/n, Universidad de Málaga, 29071 Málaga, Spain; juro@uma.es; 4 Law School, Campus Las Lagunillas. Edificio Rectorado (B1), Universidad de Jaén, 41092 Jaén, Spain; mgarciaj@ujaen.es

**Keywords:** program evaluation, experimental, intervention, olive oil mills, enforcement

## Abstract

(1) Objective: The objective of this study was to evaluate the effect of enforcement capabilities on the efficacy of two public interventions to improve occupational safety in olive mills. (2) Method: The difference in efficacy was evaluated by comparing the risks detected in two groups in an initial assessment (with visits in the 2006–2008 period) to the risks detected in a later assessment, either after an intervention by Authorized Technicians with enforcement capabilities or after an intervention by Technical Advisors without enforcement capabilities (2011–2013). The assessments identified risks in the companies, according to a specific risk map for olive oil mill enterprises. (3) Results: Statistically significant differences were observed in the comparison of the risk reductions of the two groups, with regard to the initial assessment. The greatest reductions in risk were found in the companies that received interventions by Authorized Technicians with enforcement capabilities, in the sections of individual protection equipment and the storage of dangerous products. (4) Conclusion: Interventions by Authorized Technicians with enforcement capabilities are more effective than interventions by Technical Advisors without enforcement capabilities.

## 1. Introduction

### 1.1. The Olive Oil Mills Sector

Spain, in addition to being the country with the greatest area dedicated to olive groves and the greatest number of olive trees, is the leading producer and exporter of olive oil and table olives in the world. This industrial oil sector is represented by olive mills and bottling companies (the sector that transforms the olives into oil), as well as so-called “auxiliary” industries, such as olive pulp extractors and refineries [1].

The olive grove, and the industry associated with it, represents an economic activity that, throughout its history, has been located in certain areas of Spain, particularly, in Jaén, Andalusia. The olive grove, as indicated in the Andalusian Olive Law, is the most representative and symbolic “agro-system” in Andalusia. At present, from an economic point of view, Andalusia maintains a clear global leadership in olive groves, which are an indispensable element of the social and territorial cohesion of its regions and have high environmental value. They are distributed throughout the Andalusian provinces, but acquire special importance in the province of Jaén, which is south of the province of Cordoba, northwest of Granada, north of Malaga, and southeast of Seville, all of which make up what is known as the “Andalusian Olive Grove Axis”.

With regard to the figures for olive oil mills in Spain, there are currently 1750 olive mills, according to the Olive Oil Agency, distributed throughout 13 Autonomous Communities. Andalusia has the greatest percentage, almost 47% of the total, with 816 mills. Of the olive mills located in the Autonomous Community of Andalucía (816), 323 (approximately 40%) are found in Jaén (see Table 1).

The olive oil industry in Jaén, as the largest producer of olive oil in the world (its olive oil production constitutes about 30% of the worldwide production and 80% of the national production), has important economic relevance in the province of Jaén. Therefore, the importance of these companies justifies their consideration and the fact that they are the object of analysis and study in different research fields: in this case, occupational health and safety.

Previous research has identified the main risks [3] and accident rates [4], and that most of the companies in the sector in Jaén are small and medium businesses [3,4].

### 1.2. Evaluation of Public Policies in Matters of Occupational Health and Safety: Assessment of Intervention Efficacy

The evaluation of public policies in matters of occupational health and safety is an area of growing interest [5]. Many occupational health and safety public policies include intervention programs and the evaluation of their effectiveness [6].

Occupational health and safety interventions can be classified, according to their targets, into the following main groups [7]:

- Technical interventions on the equipment and work installations;
- Organizational interventions on work procedures;
- Psychosocial interventions on the personnel, their behaviour, and training;
- Complex interventions, combining elements from the previous groups.

There are three main factors in the conceptual framework which can be used to analyse occupational health and safety interventions [8]:

- Intervention design: necessary changes, better ways to achieve them, what the barriers are, who the decision-makers are, and so on;
- Implementation: components and target group, degree of success and extent of the application of the measures, and so on;
- Evaluation of effectiveness.

Regarding the methodologies for evaluating interventions to improve occupational health and safety, the National Institute for Occupational Safety and Health (NIOSH) guide [9] stands out. It was created in the United States and includes the quality requirements and criteria for the evaluation of interventions.

As for the effectiveness of interventions to improve safety, evidence of their usefulness is difficult to gather without a proper design. A systematic review by Breslin [10] showed that well-designed evaluations can be effective.

All interventions are designed to act on certain internal processes or variables within the prevention system. If the effect on the outcomes, such as injury rates [11], is difficult to observe, an evaluation of intermediate objectives can be proposed, based on the changes expected in the processes or variables addressed [12]. This method is especially appropriate when the number of accidents is insufficient to observe significant effects [13].

The application of program theory in the evaluation of interventions to improve safety involves the identification of changes in the risk factors to be acted on and their measurement before and after the intervention [14].

One of the main types of interventions used by public authorities is control and sanctions (enforcement). To date, various studies have been conducted to evaluate public interventions with enforcement programs.

With regard to the state-of-the-art of interventions by public authorities, one of the most complete studies was commissioned by the Australian Government and carried out by WorkSafe Australia [11]. This review highlighted the following elements in an intervention by Labour Authorities [15]:

- Awareness of the requirements;
- Businesses’ understanding of what they need to do to comply;
- Concern for reputation;
- Perception of their level of risk.

Furthermore, the review identified the following elements that make an inspection with enforcement successful [15]:

- Specific deterrence—imposing sanctions deters individual businesses from repeating the offence;
- General deterrence—imposing sanctions deters further breaches by the business and by other businesses;
- Bounded rationality—suggests that sanctions have the effect of drawing the attention of managers to the issue of safety;
- Co-operation—assumes that businesses want to comply with regulations.

A number of studies have been carried out in the USA on the efficacy of the Occupational Safety and Health Administration (OSHA) inspection system. The impact of inspections has been analysed [16], finding a 9% reduction in accident rates. A cohort of companies with between 50 and 250 employees inspected by OSHA in the United States was analysed [16] and the inspected companies showed improvements in their accident rates. Recently, the re-occurrence of violations has been analysed [17].

A Cochrane systematic review [18] suggested the need for better-designed evaluations. Regarding the studies included in the review, there is evidence that inspections decrease injuries in the long term but not in the short term; however, the quality of the evidence was low to very low.

There have been two new reviews in this issue recently. The first of them indicated that public policies may reduce injuries and improve compliance [19], while the second identified strong evidence that regulatory enforcement is effective, with evidence of the effect of inspections with penalties in decreasing injuries, although consultative activity had no effect on injury outcomes in all cases [20].

It is important to highlight the difficulties and delays due to the reluctance of regulators to let researchers access data regarding their intervention programs, especially when their performance may be questioned.

### 1.3. Interventions in Small and Medium Enterprises

Most olive oil mills in Andalusia are small and medium enterprises. Intervening in small and medium enterprises (SMEs) should be a priority for public authorities, as the risks can be greater [21] and the accident rates higher [22].

Thus, it is important to adequately design interventions, taking into account the role of the safety advisors [23] and with the understanding that SME responses to regulations can be different [24].

All of this explains the fact that the recently passed European Union Strategic Framework on Health and Safety at Work 2016–2020 dedicated specific attention to these companies [25].

Recently, the journal *Safety Science* published a Special Issue on managing safety in small and medium enterprises, which included a number of studies in the field of safety interventions in SMEs [26].

In one of these studies, an injury prevention project in metalworking micro-enterprises was analysed [27]. The intervention included technical assessment by technicians without enforcement capabilities. The program was effective in improving the working environment, but there were no significant differences between enterprises that did or did not participate in meetings with the advisors.

### 1.4. Legal Regulation at the Time of the Research

In Spain, there is a clear separation of the public bodies related to health and safety at work. On one hand, labour inspectors are a national body, although they are co-ordinated by the Regional Governments. Labour Inspectors have total enforcement capacities, according to the Labour Inspection Convention of the International Labour Organization.

At the same time, each of the Regional Governments in Spain has an Institution for advice, guidance, and support of health and safety activities mandatory for enterprises. In Andalusia, this institution has, in each of the eight provinces, a Centro de Prevención de Riesgos Laborales (Occupational Risk Prevention Centre). These centres, which have been in operation since the 1970s, have been advising and visiting work establishments, employing highly qualified assessors, most of which have engineering and science university degrees. However, there is a concern that their role, with no enforcement capacity, could be a burden on their activity. At the same time, most Labour Inspectors have law degrees and lack proper knowledge of the technical issues in health and safety.

Therefore, a new regulation was established in 2005, the ROYAL DECREE 689/2005 [28], allowing technical assessors to become technical inspectors with enforcement capacities.

### 1.5. Intervention Program by Authorized Technicians: Purpose of This Research

In Spain, according to the ROYAL DECREE 689/2005 [28], technical assessors may become inspectors of working conditions with enforcement capabilities, with the denomination of Authorized Technicians. A joint Commission, which includes members of the national government and the regional government of Andalusia [29] programs their activity each year.

Regarding the evaluation of Authorized Technicians, Carrillo-Castrillo et al. [30] analysed the evolution of the enterprises which were assessed and not assessed, observing that the reduction in accidents in the assessed enterprises was higher than in the rest of the enterprises in the manufacturing sector in the period 2009–2011. However, the authors of this study remarked on the need of a control group to avoid bias of selection.

The interventions by the Authorized Technicians, as public employees of the Labour Administration, consist of verifying the material working conditions in enterprises, based on the contents established in the corresponding Service Order.

The hypothesis is that enforcement capacities have a positive effect on the risk reduction of the interventions. Thus, the main purpose of this research is to evaluate the effect of Authorized Technicians and demonstrate that the addition of enforcement capacities to the public assessor servants increases the effect of the intervention. A quantitative measurement of risk reduction is used.

## 2. Materials and Methods

### 2.1. Olive Mills Sampling

This is an experimental design study with two intervention groups, selected randomly [2]. We selected two groups of olive mills, an intervention group and a control group. In order to avoid any bias, neither of these olive mills had been visited by the Labour Inspectors in the intervention period. Therefore, the only interventions from public bodies in these olive mills were the ones analysed.

The olive mills in both groups were selected randomly using the Labour Authority databases. In these databases, 302 olive mills were available in the Jaen province. The number of olive mills in each of the groups were determined by the Labour Authority, based on the number of Assessors available and the campaign activity. It must be kept in mind that the number of days of that olive mills work depends on the climate, and so the visits need to be performed in the campaign period when the olive mills are in operation.

The number of olive mills finally visited was 43 in the intervention group and 72 in the control group. This sample was considered enough to provide statistical significance.

All olive mills had less than 50 workers and the number of workers fluctuates enormously throughout the year in the olive oil mills (which is common for this industrial activity, where 75% of the enterprises have less than 10 workers) [4]. Therefore, all of them are small enterprises according to the European Union criteria.

### 2.2. Study Design and Data Collection

The efficacy of the Authorized Technicians program (assessors with enforcement capabilities) was evaluated by comparing the effect of the intervention by technicians in a group of companies (intervention group) and the effect of the intervention by Technical Advisors without enforcement capabilities in another group of companies (control group). In order to avoid any bias, neither of these two groups included companies that had been visited by the Work and Social Security Inspection.

To collect the necessary information to assess the existing risks, an Assessment Protocol was created (see Table 2). This risk assessment protocol included the 31 most frequent risks in the olive mill sector, which has been used previously in academic research. The protocol has been explained elsewhere [2,3,4]. Data collection, thus, consisted of the identification of the risk levels according to the assessment protocol in each of the 31 possible risks. Each enterprise had its own specific risk map, based on the risks identified.

The effects of the interventions on the two groups of companies were measured by comparing the serious, very serious, and unacceptable risks detected in the companies before and after the interventions in both groups and in the control group. The effect of the intervention was measured using the risk levels arrived at based on the protocol.

The risk levels were assessed as having serious, very serious, or unacceptable levels based on the methodology presented in the NTP 330 [31]. This technical prevention rating has the same risk assessment matrix as the BS 8800 norm [32].

All of the risk assessments were performed by public inspectors from the Centro de Prevención de Riesgos Laborales de Jaén (Occupational Risk Prevention Centre of Jaén), who were highly trained in Occupational Risk Prevention. In Spain, being highly trained in Occupational Health and Safety means having a university degree and at least 650 h of post-graduate university training, according to the syllabus included in the Royal Decree 39/1997 [33].

In each Occupational Risk Prevention Centre, there are two types of inspectors: Authorized Technicians (who have enforcement capabilities) and Technical Advisors (who do not). All inspectors participating in the study met the criteria for being highly trained in Occupational Health and Safety and had at least two years of prior experience inspecting olive mills. It must be noted that olive mills are very common in Jaén and all of the public inspectors in the Occupational Risk Prevention Centre of Jaén are experts in this specific type of establishment.

This study incorporated three assessments of risk (see Table 3):

- Initial assessment of all companies in both the intervention and control groups within the 2006–2008 period. Therefore, this first evaluation took place before the interventions (before both interventions). Evaluations were made of 115 olive mills (43 from the intervention group and 72 from the control group);
- Assessment of the companies in the intervention group (i.e., companies with intervention by Authorized Technicians with enforcement capabilities) in the 2011–2013 period;
- Assessment of the companies in the control group (i.e., companies with intervention by technical advisors without enforcement capabilities) in the 2011–2013 period.

### 2.3. Measurement of the Intervention Group: Olive Mills Receiving Intervention by Authorized Technicians with Enforcement Capabilities (2011–2013)

The intervention group was composed of 43 olive mills (13.3% of the census in the province of Jaén), which received an intervention by Authorized Technicians with enforcement capabilities from the Occupational Risk Prevention Centre of Jaén in the 2011–2013 period. This group was randomly selected from the census of enterprises in the province of Jaén [2], excluding those that had been visited by the Work and Social Security Inspection in the period of under study.

The intervention by authorized technicians consisted of the following three phases:

1. Initial visit, verifying the working conditions and whether they comply with the standards;
2. Requiring the company to correct the deficiencies found and assigning a time period for their resolution (which, in any case, must not exceed six months);
3. Final visit, verifying that the deficiencies have been remedied by the end of the designated time period. If this has not been done, the technician proposes the initiation of an infraction and sanction procedure.

Assessment of the existing risk levels, both in the initial and final visits of the intervention, was carried out by the Technical Advisor with enforcement capabilities according to the Assessment Protocol (see Table 2).

### 2.4. Measurement in the Control Group: Olive Mills with No Intervention by Authorized Technicians, But in Which Intervention by Technical Advisors Did Take Place (2011–2013)

The group with an intervention by Technical Advisors without enforcement capabilities was composed of 72 olive mills (23.3% of the census). The sample for the study’s comparison group was randomly selected from all the enterprises in the census for the province of Jaén (olive oil agency, 2013), excluding those with intervention by Authorized Technicians with enforcement capabilities and those that had been visited by the Work and Social Security Inspection during the period of the study (2008–2013).

The interventions in these companies were carried out by Technical Advisors without enforcement capabilities. The intervention protocol consisted of three phases:

1. Initial visit, verifying the work conditions and their compliance with the standards;
2. Recommending that the company correct the deficiencies found and assigning a time period for their resolution (which, in any case, must not exceed six months);
3. Final visit, verifying that the deficiencies have been remedied at the end of the designated time period.

The assessment of the existing risk levels, both in initial and final visit of the intervention, was carried out by the Technical Advisor without enforcement capabilities, according to the Assessment Protocol (see Table 2).

### 2.5. Statistical Analysis

The difference in the reduction in the number of risks before and after the intervention in both groups was tested using the Chi-Square test [9]. For a specific category of work condition, the contingency table for Chi-square test was prepared following Table 4.

## 3. Results

### 3.1. Effect of the Intervention by Authorized Technicians with Enforcement Capabilities (2011–2013)

Of the 43 companies where the intervention took place, a total of 463 risks was found in the initial assessment, with 379 risks resolved after the intervention by the Authorized Technicians with enforcement capabilities; that is, 82% of the risks identified in the initial assessment were corrected.

A detailed list of the percentages of risks resolved after the intervention by the Authorized Technicians with enforcement capabilities, according to the Assessment Protocol (see Table 2), is presented in Table 5.

### 3.2. Effect of the Intervention by Technical Advisors without Enforcement Capabilities (2011–2013)

In the 72 companies visited, the initial assessment detected 912 risks, with 229 of them corrected after the intervention by the Technical Advisors without enforcement capabilities; that is, 25% of the risks found in the initial assessment were resolved.

A detailed list of the percentages of risks corrected after the intervention by the Technical Advisors without enforcement capabilities, according to the Assessment Protocol (see Table 2), is presented in Table 6.

### 3.3. Comparison of the Effects of the Two Interventions

**Figure 1:**
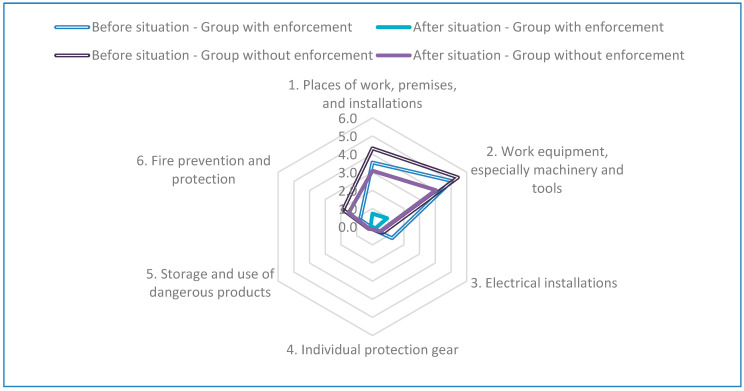
Radar chart.

The results of the Chi-square test for the contingency table of the number of risks before and after intervention by Authorized Technicians with enforcement capabilities (2011–2013) and by Technical Advisors without enforcement capabilities (2011–2013) are presented in Table 7 and Figure 1.

For the enterprises with intervention by Authorized Technicians with enforcement capabilities, the mean number of risks was 10.8 before the intervention and 2.0 after the intervention. For the enterprises with intervention by Technical Advisors without enforcement capabilities, the mean number of risks was 12.7 before the intervention and 7.4 after the intervention.

For the confidence intervals for proportion of risk corrected, in the enterprises with intervention with enforcement it was (0.72–0.94), whereas in enterprises with intervention without enforcement it was (0.07–0.30), showing the clear effect of enforcement capabilities in risk.

## 4. Discussion

In the design of evaluation methods for public intervention programs, it is difficult to use a random case-control approach. In this study, we avoided possible bias, as shown in previous attempts [30]. At the same time, this study focused on a reduced number of enterprises with a common manufacturing process and clear identification of the risks before and after.

Evaluation based on risk reduction is more program-centred than the observation of injury rates [12] and indicates the mechanisms of improvement. At the same time, as there were two interventions, with the only difference being the enforcement capacity of the technicians, this study could assess the differential effect of enforcement in these interventions.

To date, this is the first study that has analysed the effect of Authorized Technicians in Spain and the benefits of enforcement capabilities. It was assumed that Authorized Technicians were a useful tool for interventions in Small and Medium Enterprises but, until this research, no evidence has been given regarding the effect of their effectiveness. This regulation of enforcement in technical advisors can provide a useful approach in those countries that have such advisory institutions but which do not include enforcement capabilities. At the same time, these Authorized Technicians are experts on occupational health and safety and can better assess safety risks than labour inspectors, which are law and regulation experts but not technical ones.

Previous reviews [10,18,19,20] have not evaluated the effect of enforcement, as in this study. Additionally, most previous studies have evaluated effectiveness in injury reduction, whereas, in this study, a risk assessment approach was used.

In the 43 olive mills visited by Authorized Technicians with enforcement capabilities in the period 2011–2013, a total of 463 risks were initially found. In 25 companies, the requirements were fully addressed, meaning that all 295 risks were eliminated in these firms.

In the remaining 17 enterprises, 168 risks were initially detected, of which 83 requirements were resolved, leaving 87 risks (approximately 19% of the initial total of 463 risks). These 87 risks led to a report to Work Inspection, which was not involved in the analysed intervention.

The Chi-square test was used to show the significant associations in each of the risk groups. Enterprises with intervention with enforcement had higher risk reduction than those with intervention without enforcement.

Therefore, the “efficacy” of the intervention by Authorized Technicians with enforcement capabilities was above 80% (375 risks eliminated). Consequently, intervention by technicians with enforcement capabilities produced a significant improvement in the material working conditions of the companies incorporated in the Authorized Technician program.

It is worth noting that both Authorized Technicians and Technical Advisors belonged to the same organization, had the same training and expertise, and used the same assessment approach. The only difference was that one had enforcement capabilities and the other did not.

If we compared the 80% efficacy of the Technicians to the data extracted from the interventions carried out by the Technical Advisors without enforcement capabilities in the 2012–2013 period, where only 25% of the recommendations were followed, we can observe that having enforcement capability considerably increases the intervention’s efficacy. Therefore, evidence was found that the enforcement capabilities of the inspectors increased the effect of their interventions.

This is the first published study to use an intervention protocol to determine the differential effect of an intervention with and without enforcement. The differences found, at least in the case of the Jaén olive mills, indicate that the enforcement capability of technicians plays an important role in the final result of the intervention.

Regarding the study limitations, the first thing to keep in mind is that the results obtained refer to specific programs in the region of Andalusia. In other territories with different intervention designs, the results might not be the same. Another limitation is that, in the experimental study design, although control and intervention groups were randomly selected, there was no intervention group without any intervention to assess the baseline effect.

## 5. Conclusions

The main conclusion of this study is that the public efficacy of an intervention was increased when the technical advisor had enforcement capabilities. This should lead labour authorities to consider the need to give coercive power to technicians from different agencies concerned with occupational health and safety.

In addition, the importance of the effect found recommends intensifying and increasing the number of interventions by authorized technicians in olive mills in Jaén and, in general, in the sectors of interest in Andalusia.

In these interventions and their evaluation, it is necessary to consider protocols based on risk maps, such as those proposed in this study, as they make it possible to test the efficacy of the interventions carried out.

## Figures and Tables

**Table 1:** Number of olive mills in Andalusia [2].

Province	Total	% of Andalusia
Almería	25	3.06
Cádiz	14	1.72
Córdoba	182	22.3
Granada	109	13.4
Huelva	17	2.08
Jaén	323	39.6
Málaga	67	8.21
Sevilla	79	9.68
Total general	816	100

**Table 2:** Assessment Protocol for the most frequent irsks in olive mills (Source: created by the authors).

Working Conditions	Risk Code	Risk Description	Frequency
1. Places of work, premises, and installations	1.1	Stepping on objects	
	1.2	Falls on the same level	
	1.3	Falls to a lower level	
	1.4	Crashing into moving objects	
	1.5	Falling loose objects	
	1.6	Insufficient lighting	
	1.7	Exp. to adverse climatic conditions	
	1.8	Exposure to noise	
	1.9	Risk of physical fatigue	
	1.10	Exposure to Chemical agents	
	1.11	Exposure to Biological agents	
	1.12	Exposure to oxygen-deficient atmosphere	
	1.13	Projection of particles	
2. Work equipment, especially machinery and tools	2.1	Blows/cuts with objects and tools	
	2.2	Being trapped by or between objects	
	2.3	Over-exertion	
	2.4	Crashing into moving objects or being run over.	
	2.5	Contact with heat	
3. Electrical installations	3.1	Exposure to electrical risk	
4. Individual protection gear	4.1	Exposure to noise	
	4.2	Contact with heat	
	4.3	Mechanical contact	
	4.4	Electrical contact	
	4.5	Exposure to Chemical agents	
	4.6	Exposure to Biological agents	
	4.7	Exposure to oxygen-deficient atmosphere	
5. Storage and use of dangerous products	5.1	Exposure to Chemical agents	
	5.2	Risk of fire	
	5.3	Risk of explosion	
6. Fire prevention and protection	6.1	Risk of fire	
	6.2	Risk of explosion	

**Table 4:** Contingency Table for each specific Work Condition (or for all of them), where n_ab_ is for the number of risk of each group and period.

Period/Group	Intervention Group	Control Group	Total
Before Intervention	n_i1_	n_c1_	n_i1_ + n_c1_
After intervention	n_i2_	n_c2_	n_i2_ + n_c2_
Total	n_i1_ + n_i2_	n_c1_ + n_c2_	n_i2_ + n_i2_ + n_c1_ + n_c2_

**Table 5:** Results of the intervention by Authorized Technicians (with enforcement).

Work Conditions	Risks Detected	Risks Corrected	Percentage Corrected
1. Places of work, premises, and installations	152	121	80%
2. Work equipment, especially machinery and tools	217	177	82%
3. Electrical installations	54	46	85%
4. Individual protection gear	6	6	100%
5. Storage and use of dangerous products	3	2	67%
6. Fire prevention and protection	34	29	85%
TOTAL	466	381	82%

**Table 6:** Results of the intervention by Technical Advisors (without enforcement).

Work Conditions	Risks Detected	Risks Corrected	Percentage Corrected
1. Places of work, premises, and installations	311	89	29%
2. Work equipment, especially machinery and tools	391	101	26%
3. Electrical installations	48	12	25%
4. Individual protection gear	11	0	0%
5. Storage and use of dangerous products	17	2	12%
6. Fire prevention and protection	134	25	19%
TOTAL	912	229	25%

**Table 7:** Chi-square test of Authorized Technicians (with enforcement) and Technical Advisors (without enforcement) and percentages of corrected risks.

Work Conditions	Chi-Square *p*-Value	Percentage of Corrected Risks	
		Authorized Technicians (with Enforcement)	Technical Advisors (without Enforcement)
1. Places of work, premises, and installations	<0.001	80%	29%
2. Work equipment, especially machinery and tools	<0.001	82%	26%
3. Electrical installations	<0.001	85%	25%
4. Individual protection gear	<0.001	100%	0%
5. Storage and use of dangerous products	<0.001	67%	12%
6. Fire prevention and protection	<0.001	85%	19%
TOTAL	<0.001	82%	25%

**Table 3:** Intervention study design.

Period	Olive Mills	Measurements	Intervention	Results
2006–2008	All	Risk Assessment	General advice, no enforcement	Risk map
2011–2013	Intervention group	Working Conditions	Requirements, with enforcement	Risk reduction
2011–2013	Control group	Working Conditions	Requirements, no enforcement	Risk reduction

## Glossary

Authorized Technicians: Public employees that assess safety compliance with enforcement capabilities. Technical Advisors: Public employees that advise enterprises for safety improvements without enforcement capabilities.
